# Analysis of proton exchange membranes for fuel cells based on statistical theory and data mining

**DOI:** 10.1016/j.isci.2024.109360

**Published:** 2024-03-02

**Authors:** Hong Wang, Liang Yang

**Affiliations:** 1 School of Physics and Electronic Information, Yan’an University, Yan’an 716000, China

**Keywords:** Membranes, Energy Modeling

## Abstract

Fuel cells (FCs) have attracted widespread attention as a highly efficient, clean, and renewable energy conversion technology. Proton exchange membrane (PEM), as one of the core components of FCs, plays a crucial role, and a comprehensive summary of its development is essential for promoting rapid progress in the field of sustainable energy. This article provides a comprehensive review of the development status and research trends of PEMs over the past twenty-eight years, based on statistical analysis and data mining techniques. Price, sustainability, stability, and compatibility issues are the main challenges faced by current PEMs used in FCs research. The current research focuses mainly on the characterization, performance optimization, enhancement mechanisms, and applications of PEMs in FCs. This review provides a systematic summary of PEM materials, serving as a valuable reference for the development, application, and promotion of new PEM materials in FCs.

## Graphical abstract

**Figure:**
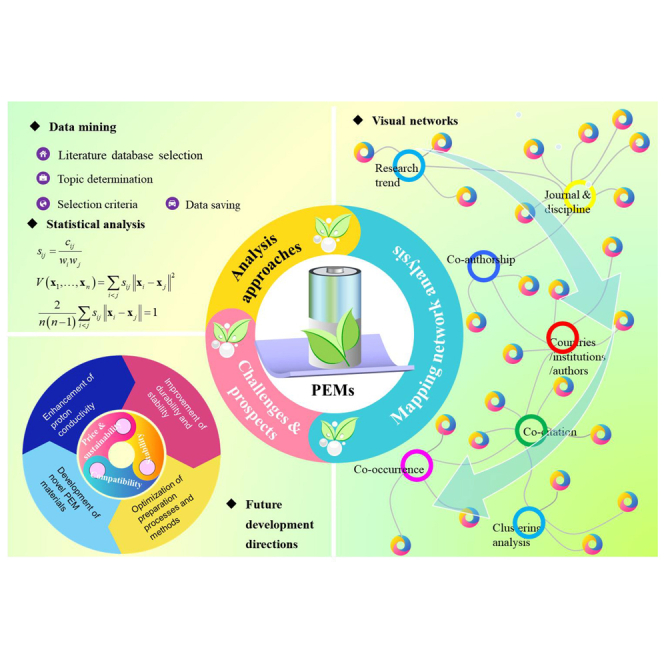

## Introduction

**Figure 1:**
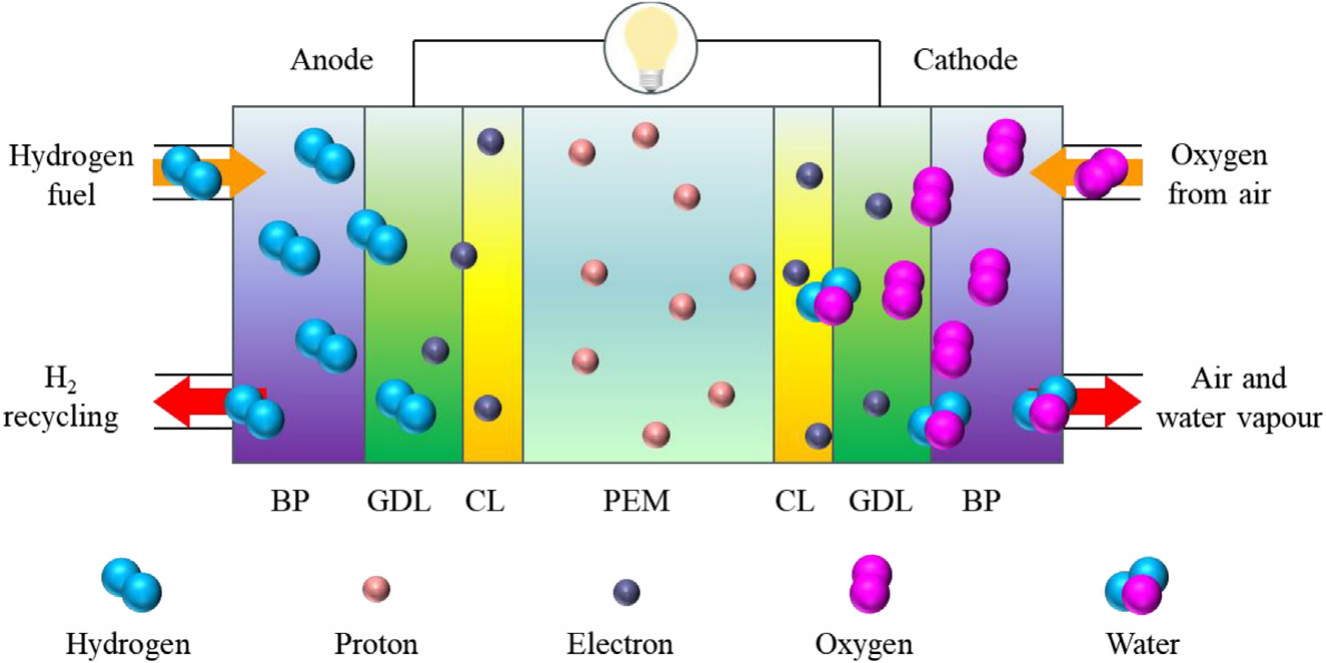
Structure diagram of PEMFC

With the continuous increase in global population and sustained economic development, energy scarcity and environmental pollution have become the main contradictions restricting economic and social sustainable development.^1^^,^^2^^,^^3^^,^^4^^,^^5^ Fuel cells (FCs) are efficient, clean, and renewable energy conversion technologies that can directly convert chemical energy into electricity, producing no emissions and being environmentally friendly.^6^^,^^7^^,^^8^ FCs have advantages such as high energy density, rapid startup, flexibility, and reliability, making them suitable for various applications,^9^^,^^10^^,^^11^^,^^12^^,^^13^ including transportation, portable power sources, power supply, and industrial applications. Additionally, FCs can be combined with other energy technologies,^14^^,^^15^ such as solar and wind energy, to form hybrid energy systems, further improving energy utilization efficiency and sustainable development. Proton exchange membrane fuel cells (PEMFCs) are typical representatives of FCs^16^^,^^17^ and have many outstanding performances.^18^^,^^19^^,^^20^^,^^21^^,^^22^ They are considered one of the most promising sustainable power generation technologies globally.^23^^,^^24^^,^^25^ The PEMFCs consist of multiple layers,^26^^,^^27^^,^^28^^,^^29^ with a proton exchange membrane (PEM) in the middle, catalyst layers on both sides, and gas diffusion layers further outward. These five layers form a membrane electrode assembly, with bipolar plates on both sides of the membrane electrode assembly, as shown in Figure 1. The electrochemical reactions occur in the PEMFC by introducing hydrogen gas at the anode for oxidation and oxygen or air at the cathode for reduction, generating water with the assistance of catalysts. The reactions occurring in the PEMFC can be explained as follows:

(Equation 1) $${\text{Anode}:H}_{2}\to 2H^{+}+2e^{-}$$

(Equation 2) $$\text{Cathode}:\frac{1}{2}\;O_{2}+2H^{+}+2e^{-}\to H_{2}O$$

(Equation 3) $${\text{Overall}:H}_{2}+\frac{1}{2}\;O_{2}\to H_{2}O$$

Most current research works are primarily focused on FCs themselves, membrane electrodes, anode catalyst layers, and catalyst materials.^30^ The PEMs are one of the key components of PEMFCs, and their critical role cannot be overlooked.^31^ They separate hydrogen and oxygen gases in the electrochemical reaction and facilitate proton conduction, resulting in the generation of electric current.^32^^,^^33^ The PEMs effectively provide proton conduction pathways while preventing electron transport, thereby enabling the normal functioning of FCs. Commonly used PEMs include perfluorosulfonic acid membranes,^34^^,^^35^^,^^36^^,^^37^ poly(ether ether ketone)s (PEEKs),^38^^,^^39^ polyimides (PIs),^40^^,^^41^ polybenzimidazoles,^42^ polyphenylsulfone,^43^^,^^44^ and poly(arylene ether)s.^45^^,^^46^^,^^47^ These membranes find wide applications, particularly in FC vehicles and portable electronic devices.^48^ FC vehicles, as sustainable transportation solutions,^49^ heavily rely on PEMFCs.^50^^,^^51^ The high proton conductivity and stability of PEMs are crucial for the performance and reliability of FC vehicles.^52^ Additionally, PEMs can be used in portable electronic devices^53^^,^^54^ such as mobile phones and laptops to provide sustainable power sources. To enhance the efficiency, stability, and reliability of FCs, in-depth research on PEMs is necessary. The performance of PEMs directly affects the output power and lifespan of FCs. Therefore, by gaining a deep understanding of the structure and properties of PEMs, their design and preparation methods can be improved to enhance proton conductivity and stability.^55^ Furthermore, studying PEMs can also explore new materials and technologies to address challenges in the field of FCs. Simultaneously, optimizing the fabrication processes and methods of PEMs can reduce costs, improve production efficiency, and promote the commercial application of these membranes.

**Figure 2:**
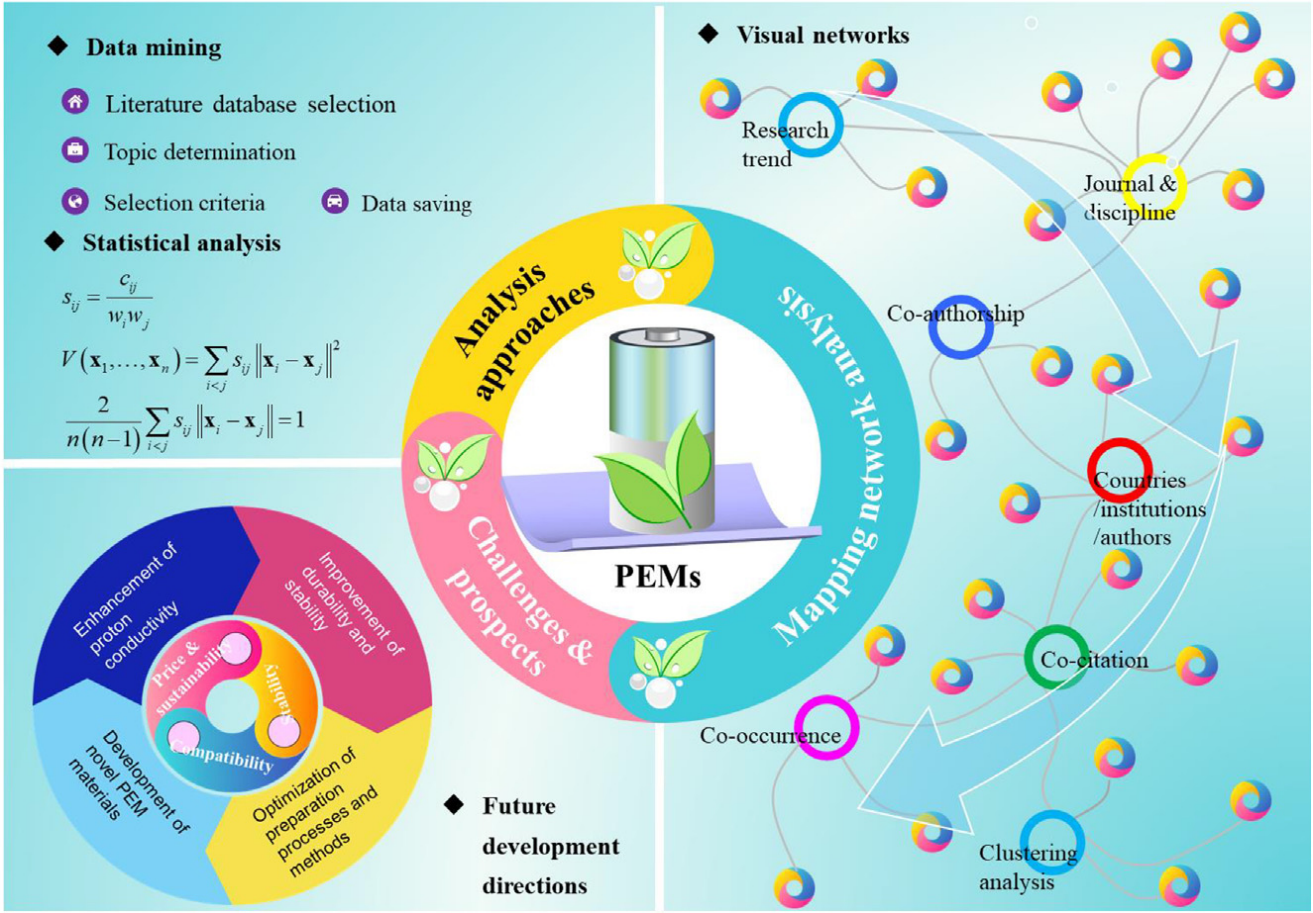
Research methodology map of this paper

Based on this, the overall overview of research on PEMs for FCs is shown in Figure 2. This study provides a systematic bibliometric analysis of PEM-FC literature over the past 28 years, aiming to identify the background and frontiers of PEM-FC research. In contrast to existing literatures,^56^^,^^57^^,^^58^ this article emphasizes using mapping networks to characterize the intrinsic relationships of PEM research, revealing the multiple factors that need to be considered in developing high-performance PEMs. To distinguish between “proton exchange membranes for fuel cells” and “proton exchange membrane fuel cells,” this article defines their abbreviations as PEM-FC and PEMFC, respectively. The research work in this article is expected to arouse strong interest among researchers in the field of energy, particularly FCs, and provide answers and insights to the following questions.

(1) What is the status and background of PEM-FC research globally?
(2) Which countries or institutions have authors who are focusing on or researching PEM-FC?
(3) Who are the authoritative or leading researchers in the field of PEM-FC?
(4) Which journals have published research results related to PEM-FC? Which journal is the most important in this field?
(5) What are the most recognized and classic articles in the field of PEM-FC research?
(6) What are the current focuses and hot topics in PEM-FC research?
(7) What are the main challenges and possible strategies in PEM-FC research? What are the potential challenges and response measures?
(8) What are the future trends and possible scenarios for PEM-FC research?

These questions will be addressed and discussed in this article, providing valuable insights into the current state and prospects of PEM-FC research.

## Analysis approaches

**Figure 3:**
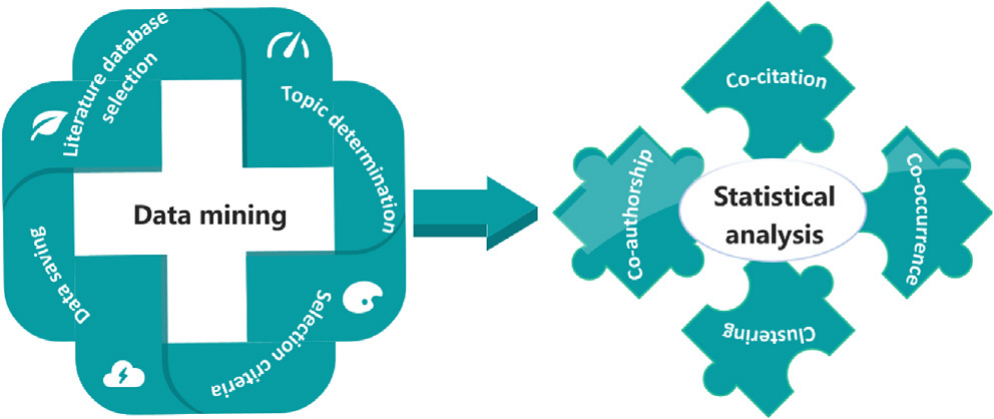
Schematic diagram of the process of analysis approaches

To investigate the research and application of PEM-FCs, this study employs a comprehensive literature data mining and statistical analysis approach to collect, assess, and analyze publications from January 1, 1995 to December 4, 2022. The process of the analysis method is illustrated in Figure 3. A comprehensive literature review is a systematic and effective approach to identify, evaluate, and summarize the research work conducted by researchers.^59^ The summary and analysis methods in this study mainly include data mining and statistical analysis. Firstly, a search is conducted in authoritative databases that record literature, and relevant published papers are selected. During the process of article screening and review, key research topics are specified, selection criteria for the studies are determined, the collection of relevant published works is evaluated, and records of the literature data are kept. Subsequently, after the systematic literature selection, the selected literature is subjected to statistical analysis, such as publication trends, authors, countries, journals of publication, co-occurrence of keywords, and cluster analysis. Finally, the shortcomings and gaps in the current research on PEMs in FCs are identified, and the main directions and opportunities for future research are proposed.

### Data mining

#### Literature database selection

Web of Science (WOS) database integrates academic journals, invention patents, conference proceedings, free web resources, and other important academic information published by information institutions. It provides academic information in various fields such as natural sciences, social sciences, academic monographs, research funding, arts, and humanities. It is considered one of the most representative authoritative databases. Therefore, this article selects the Core Collection database in WOS to search for relevant articles on the application of PEMs in FCs. This allows us to find many representative literatures closely related to the topic of this review.

#### Topic determination

To obtain more accurate literature data on PEM-FC research, this article sets the following key topic word rules.

(1) Search source setting: title, abstract, author keywords, and Keywords Plus.
(2) Breaking the restrictions of search terms and using synonyms interchangeably: words like "for," "in," and "of" are used to represent "used in."
(3) Topic word relationships: the use of Boolean logical operators and truncation symbols.

Based on this, the topic words determined in this article are ("proton exchange membrane∗ for fuel cell∗") or ("proton exchange membrane∗ in fuel cell∗") or ("proton exchange membrane∗ of fuel cell∗") or ("proton exchange membrane∗ on fuel cell∗") or ("proton exchange membrane∗ used in fuel cell∗").

#### Selection criteria

Based on the research motivation and research questions in introduction, the selection criteria for this article are as follows.

(1) The publication date of the selected papers is from January 1, 1995, to December 31, 2022. The search was conducted on December 4, 2022. It is important to note that, based on the topic determination, the first relevant paper was published in 1995.^60^
(2) Document types such as meeting abstracts, proceeding papers, book chapters, and corrections are excluded.
(3) Only articles in English are selected, and articles in other languages are excluded.
(4) The focus is on PEMs, not just FC-related papers.
(5) Papers on the use of PEMs for other applications are excluded.
(6) Research papers on improved PEM-FCs are included.

#### Data saving

Based on the topic determination and selection criteria, the relevant literature data on PEM-FCs are saved. The record content includes full records and cited references. These data are crucial for subsequent statistical analysis and provide the original basis for scientometric analysis.

### Statistical analysis

Through the aforementioned method, many literatures have been downloaded and relevant information within the articles has been extracted. Subsequently, statistical analysis is conducted to summarize certain indicators in the field, presenting various changes in the research field in the form of data and charts, such as development history, current research status, and research hotspots. Statistical methods enhance the research significance of the topic to some extent. VOSviewer is a literature mapping visualization software^61^^,^^62^^,^^63^ used to construct various networks, such as citation, bibliographic coupling, institutional collaboration, co-citation, or co-authorship relationships.^64^ It focuses on the visualization of scientific knowledge and provides text mining capabilities to construct and visualize co-occurrence relationships of important terms (keywords) extracted from many scientific literatures.^65^ Therefore, this article conducts statistical analysis of the literature on PEM-FCs using VOSviewer.

In the statistical analysis of the literature, relationships such as co-authorship, co-citation, co-occurrence, clustering of authors, national collaborations, keyword co-occurrence, and bibliographic co-citation can be represented by circles and lines, where circles represent the objects of our study and lines represent the associations between them. To avoid repetition, this article focuses on the analysis of keyword mapping. Keywords represent the core content of the research, and, in the analysis of keyword co-occurrence, analyzing the distribution of keywords can effectively reflect the research hotspots in the field.^66^ When two identical keywords are found in the selected papers, they are considered as a co-occurrence. Based on the co-occurrence matrix, a mapping graph of the literature is constructed, and the most critical step is to calculate the similarity matrix and map the similarity matrix. The co-occurrence matrix is normalized, and the similarity measurement is represented by the strength of association. Assuming two keywords can be represented as *i* and *j*, their similarity $s_{ij}$ can be expressed as

(Equation 4) $$s_{ij}=\frac{c_{ij}}{w_{i}w_{j}}$$

where $c_{ij}$ represents the number of links between keyword *i* and keyword *j*, and $w_{i}$ and $w_{j}$ represent the total number of link occurrences where keywords *i* and *j* appear together. In the mapping graph, the distance between keyword *i* and keyword *j* reflects their similarity. A shorter distance indicates a higher similarity, while a longer distance indicates a lower similarity. The idea of cluster analysis is to minimize the weighted sum of squared Euclidean distances between all pairs of items,^61^ which can be solved by minimizing the value of *V* in the following Equation 2:

(Equation 5) $$V\left(x_{1},\ldots ,x_{n}\right)={\sum_{i<j}s_{ij}\left\|x_{i}-x_{j}\right\|}^{2}$$

(Equation 6) $$\frac{2}{n\left(n-1\right)}\sum_{i<j}s_{ij}\left\|x_{i}-x_{j}\right\|=1$$

where $x_{i}$ represents the position of the keyword in the mapping graph, *n* represents the number of items in the mapping, and ||⋅|| represents the Euclidean norm. The higher the similarity between two keywords, the higher is their weight.

## Results and discussion

PEMs play a significant role in FCs. Based on the research methodology described in the analysis approaches section, a comprehensive exploration of the literature data on PEMs used in FCs over the past 28 years was conducted, resulting in a total of 301 published papers. This section will provide a systematic statistical analysis of the publication papers, authors, institutions, countries, academic journals, research fields, and research hotspots.

### Trends in the research literature

**Figure 4:**
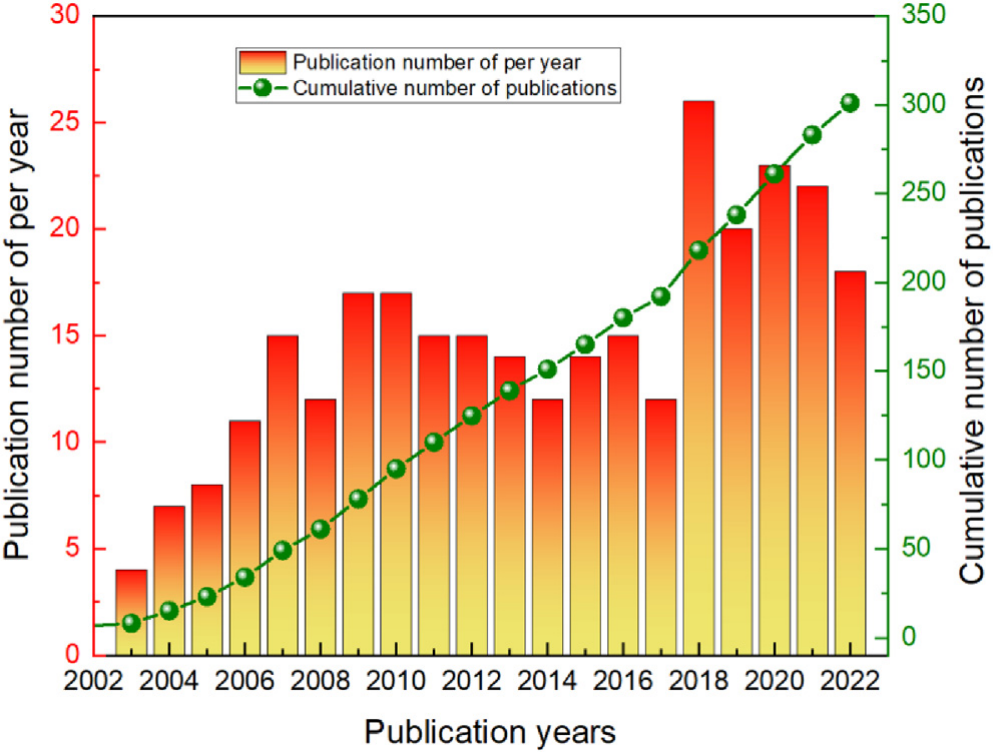
Trend of scientific and technical papers published on PEM-FCs from 1995 to 2022

Figure 4 illustrates the research output in the field of PEM-FCs from 1995 to 2022. It can be observed that, from 1995 to 2002, only four relevant research papers were published, indicating a relatively low level of interest and recognition in the research value of PEM-FCs during this period, which can be referred to as the initiation phase. In the following years, a growing interest in PEMs for FCs emerged, and, by 2009, the number of papers reached 19, representing an increase of 15 papers compared to 2003, nearly quadrupling the research output. This phase can be referred to as the growth period. After the initiation and growth phases, an average of 15.75 papers were published annually from 2010 to 2017, indicating a relatively stable development period. In 2018, the number of papers suddenly jumped to a higher level, reaching a peak of 26, which is the highest value in recent years. Subsequently, these years can be defined as the second development phase. After experiencing two rapid development phases, the topic of PEM-FCs has gained widespread attention among researchers, and it is believed that, in the coming years, continuous development and significant research achievements will be made. It should be noted that the relatively low number of published papers in 2022 is due to the absence of data for December (the search for this topic was conducted on December 4, 2022, and the database displayed the last updated data as November 30, 2022).

### Journal and discipline

Analyzing the sources of publications is an effective method to reveal the influence of academic journals. By comprehensively analyzing various indicators of publications, it is possible to dynamically track the dissemination of academic journals worldwide and periodically assess the most influential academic journals, providing a new reference dimension for evaluating the social impact of journals. In this study, publication quantity ratio, average citation count, impact factor, article influence score, and scientific fields were used as evaluation indicators to analyze the sources of publications on PEM-FC. A total of 301 papers were included in 119 academic journals, and Table 1 shows the relevant information of the top 15 active journals, which account for 50.17% of the total publications. There are six journals with more than 10 papers published, which are considered active journals in PEM-FC research. Among them, the journal "Journal of Membrane Science" dominates the importance of this research to a great extent, with 32 papers and a percentage of 10.63%. It is followed by "International Journal of Hydrogen Energy" (16, 5.32%) and "Journal of Applied Polymer Science" (16, 5.32%). In terms of average citation count, "Macromolecules" (139.19) ranks first with an absolute advantage, followed by "Polymer" (69.78), "Electrochimica Acta" (67.17), "Fuel Cells" (66.33), and "Journal of Membrane Science" (50.56). However, according to the citation indicator in 2021, the top three journals are "Journal of Materials Chemistry A" (1.85), "Journal of Membrane Science" (1.84), and "Journal of Power Sources" (1.44). In terms of impact factor and article influence score, "Journal of Materials Chemistry A" (14.511, 2.094) is the most active and influential journal, followed by "Journal of Power Sources" (9.794, 1.423) and "Journal of Membrane Science" (10.53, 1.089). It is worth noting that the article influence score is also an evaluation indicator, which normalizes the eigenfactor score according to the cumulative size of the cited journal across the prior five years. For example, an article influence score greater than 1.00 for Journal of Materials Chemistry A indicates that each article in the journal has above-average influence. Different journals have different dissemination power and social impact, and different indicators yield different evaluation results for journals. Therefore, these indicators need to be combined and further research is needed to optimize the current evaluation system.

**Table 1:** Sources of relevant publications on PEM-FC research

Journal title	Number of papers	Percentage/%	Total cites	Citations per paper	Impact factors (2021)	Citation indicator (2021)	Article influence score
Journal of Membrane Science	32	10.631	1618	50.56	10.53	1.84	1.089
International Journal of Hydrogen Energy	16	5.316	474	29.63	7.139	0.95	0.724
Journal of Applied Polymer Science	16	5.316	262	16.38	3.057	0.61	0.347
Journal of Polymer Science Part A Polymer Chemistry	11	3.654	540	49.09	2.869	0.59	0.428
Macromolecules	11	3.654	1531	139.19	6.057	1.35	1.08
Journal of Power Sources	10	3.322	374	37.40	9.794	1.44	1.423
Polymer	9	2.99	628	69.78	4.432	1	0.58
Journal of Materials Chemistry A	8	2.658	331	41.38	14.511	1.85	2.094
RSC Advances	7	2.326	134	19.14	4.036	0.57	0.519
Electrochimica Acta	6	1.993	403	67.17	7.336	1.18	0.888
Fuel Cells	6	1.993	398	66.33	2.948	0.36	0.543
European Polymer Journal	5	1.661	191	38.20	5.546	1.05	0.662
Journal of The Electrochemical Society	5	1.661	483	96.60	4.371	0.75	0.749
Membranes	5	1.661	30	6	4.562	0.61	0.589
Materials	4	1.329	56	14	3.748	0.62	0.541

**Figure 5:**
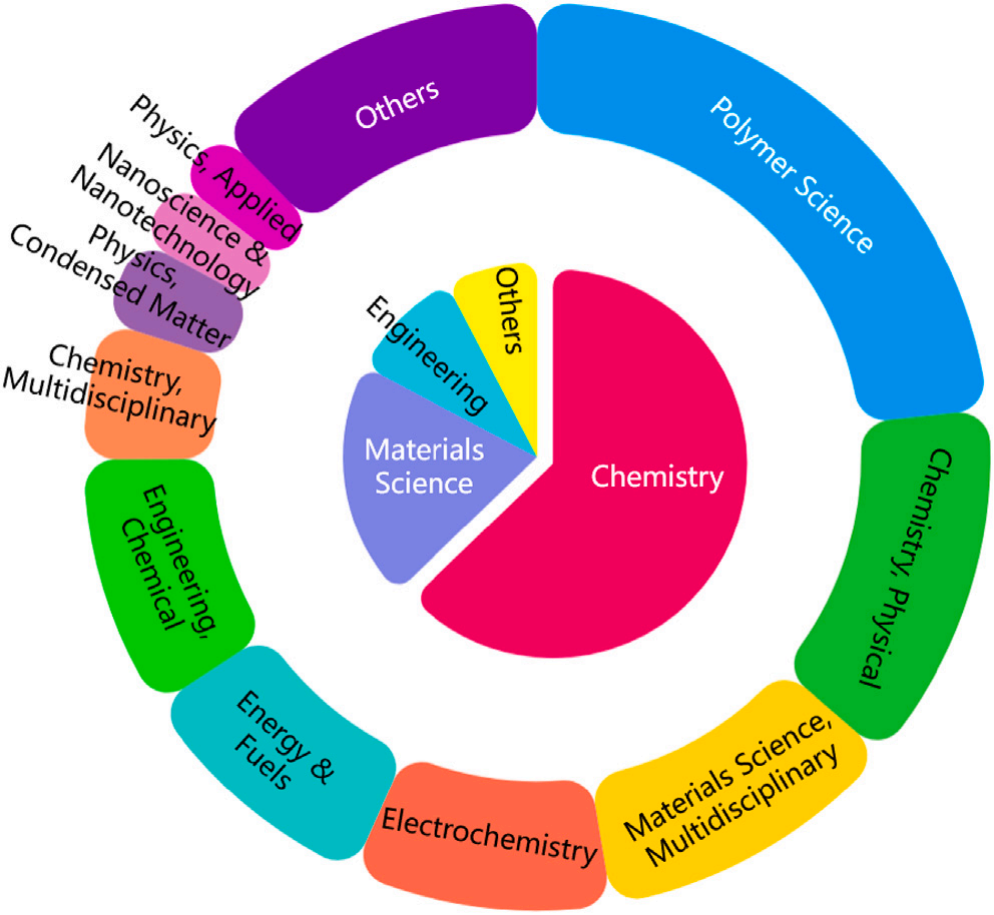
Distribution of relevant disciplines in PEM-FC research

Essential Science Indicators (ESI) is a fundamental analysis and evaluation tool for measuring scientific research performance and tracking scientific development trends based on literature records collected in the WOS database. In this study, based on ESI, the publication journals of PEM-FC research were analyzed in terms of related scientific disciplines. Figure 5 shows the statistical analysis of the 301 papers in different scientific disciplines. From the figure, it can be observed that the research on PEM-FC is mainly distributed in three category groups: chemistry (189, 62.79%), materials science (60, 19.93%), and engineering (29, 9.63%). Compared to the other two categories, chemistry shows a very active phenomenon, indicating that researchers have paid great attention to the study of PEM-FC in the field of chemistry. Further analysis of the discipline classification reveals that the research on PEM-FC mainly covers 11 disciplines, with polymer science (44.19%) being the most active field among all disciplines, which is closely related to the polymeric properties of PEM. Researchers are enthusiastic about exploring its intrinsic characteristics.

### Co-authorship analysis

Collaborative analysis can help researchers understand the academic relationships and contributions among different countries, institutions, and scholars. It is crucial for gaining insights into the development trends and academic influence of scholarly research. This section will provide a distribution and collaborative network analysis of the countries, institutions, and authors of PEM-FC papers.

#### Country distribution and collaborative network

**Figure 6:**
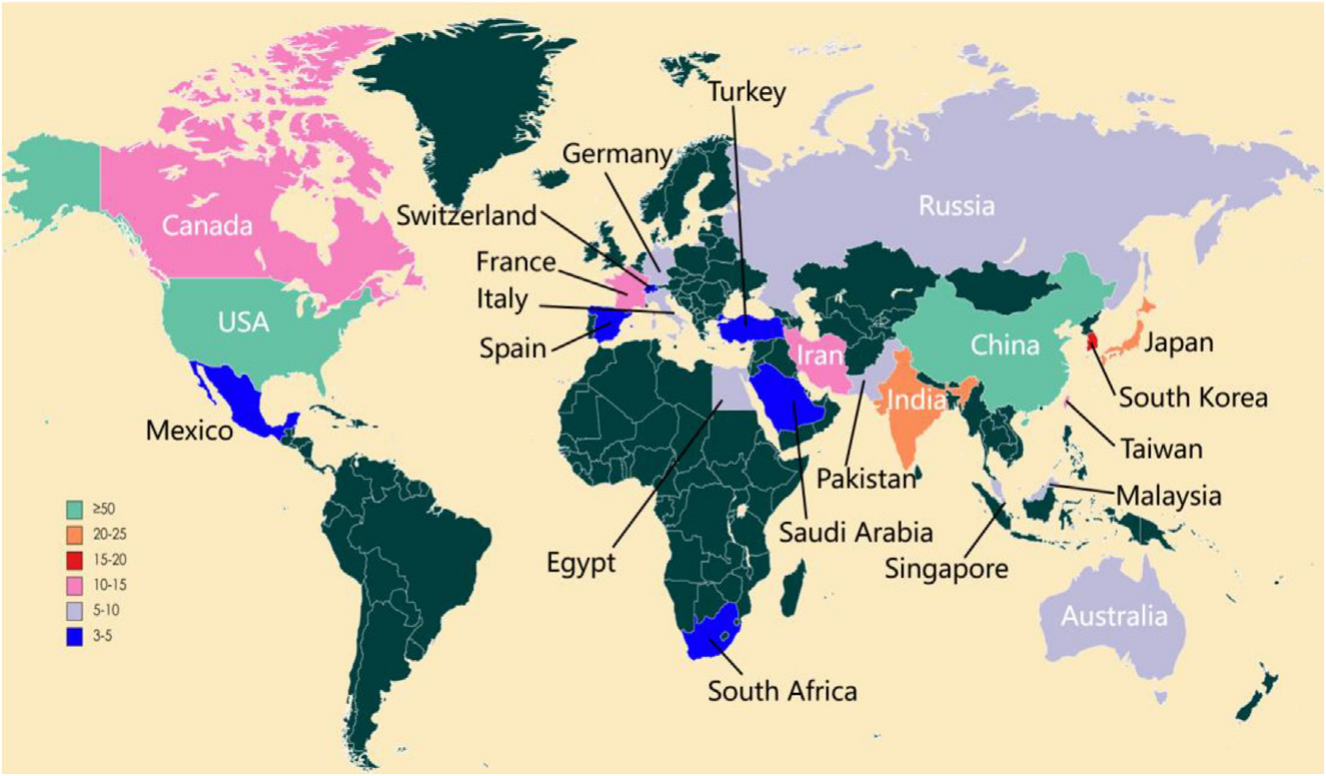
Geographic distribution of papers on PEM-FC in different countries

An analysis of PEM-FC-related papers was conducted based on the geographical locations of the published papers and authors to determine the contributions and distribution proportions of different countries. Out of the 301 literature sources obtained earlier, it was found that there were 48 countries involved. Figure 6 displays the distribution of these papers across different countries. The different colors represent the top 23 countries in terms of publication volume, accounting for 47.92% of the total number of countries. Countries with a publication count of more than 10 papers are primarily distributed in Asia (61.79%) and North America (20.27%), possibly due to the higher consumption of hydrogen energy in these regions. Africa, on the other hand, only has one country, South Africa, with a publication count of only 3 papers (1%). China (95 papers, 31.56%) has the highest number of PEM-FC-related publications among all countries, followed by the United States (50, 16.61%), India (24, 7.97%), and Japan (23, 7.64%).

**Figure 7:**
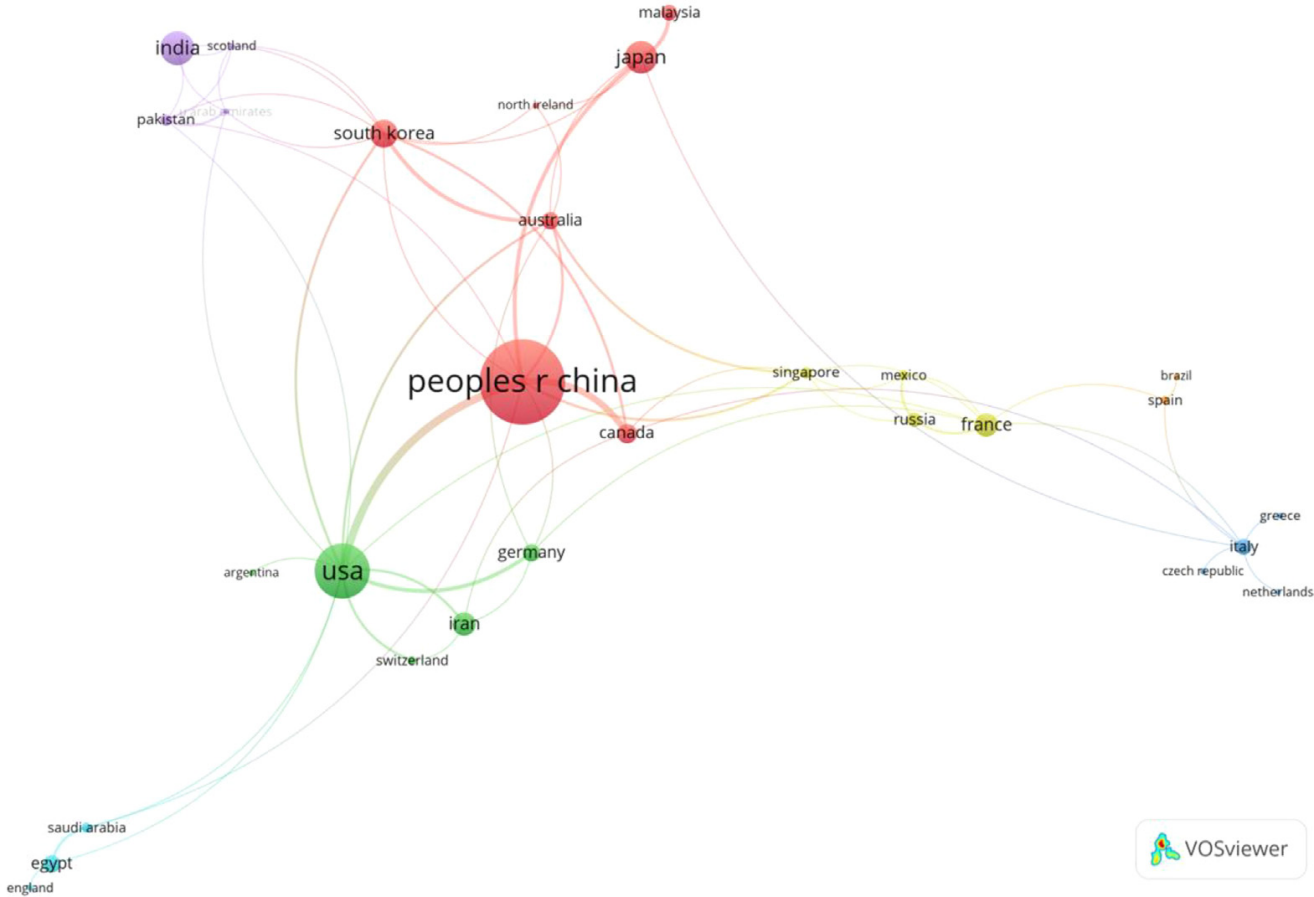
Network map of national partnerships for the study of PEM-FC

Evaluating the scientific research capabilities of countries through the construction of a collaborative network is an effective visualization method. Based on this, this study conducted a collaborative network analysis of the countries involved in PEM-FC research to provide some guidance for countries interested in this research topic. Figure 7 presents the collaborative network graph among different countries, where the size of the nodes represents the publication volume of each country, the colors represent different clusters, and the width of the lines represents the strength of collaboration between countries. From the graph, it can be observed that China has a strong collaborative relationship with other countries in the field of PEM-FC, especially with the United States. As shown in Figure 7, the collaborative exchanges among the 29 countries will promote the development of the global economic market. In the future, this strong collaborative effort will continue to promote peace and stability worldwide, while ensuring energy diversification, security, and cleanliness. With the commercialization of FCs, the market prospects are vast. FCs will become the fourth generation of power generation after thermal power, hydropower, and nuclear power, triggering a green revolution in new energy and environmental protection. However, at present, there are relatively few countries involved in PEM-FC research, and collaboration between countries is also lacking. Therefore, further strengthening international cooperation research on the main components of FCs, such as PEMs, is needed in the future.

#### Institutional distribution and institutional cooperation networks

Through statistical analysis, it was found that the 301 papers on PEM-FC research were mainly from 375 academic institutions. Table 2 shows the main academic institutions according to the number of published papers. These core institutions mainly come from China (71 publications, 12.33%), the United States (9 publications, 1.56%), Japan (8 publications, 1.39%), and Russia (7 publications, 1.22%). In terms of article citations, Jilin University ranked first with a total citation count of 1,391, followed by the National Research Council Canada (941) and Virginia Polytechnic Institute & State University (811). However, in terms of unit citation, the number of papers published by the National Research Council Canada and Virginia Polytechnic Institute & State University is not particularly prominent, but their average citation counts reached 188.20 and 166.20, respectively, which are roughly three times that of Jilin University, which has the most publications. This indicates that their academic achievements have received attention from scholars around the world and have had a positive impact on society. In terms of link numbers, the Chinese Academy of Sciences has the most links with 24, indicating that it has the most cooperation with other institutions in PEM-FC research. Jilin University, the Russian Academy of Sciences, and Sun Yat-sen University have 11 links each.

**Table 2:** Academic institutions with the highest number of published papers on PEM-FC

Ranking	Institutions	Countries	Number of documents	Proportion/%	Total citations	Citations per papers	Total link strength
1	Jilin University	China	22	3.82	1381	62.77	11
2	Chinese Academy of Sciences	China	16	2.78	441	27.56	24
3	Tokyo Institute of Technology	Japan	8	1.39	394	49.25	5
4	Russian Academy of Sciences	Russia	7	1.22	39	5.57	11
5	Dalian University of Technology	China	6	1.04	136	22.67	3
6	Iran University Science & Technology	Iran	6	1.04	82	13.67	2
7	Sun Yat-sen University	China	6	1.04	249	41.50	11
8	Universiti Teknologi Malaysia	Malaysia	6	1.04	84	14.00	6
9	Wuhan University of Technology	China	6	1.04	204	34.00	10
10	Nanjing University of Science & Technology	China	5	0.87	73	14.60	4
11	National Research Council Canada	Canada	5	0.87	941	188.20	5
12	Shanghai Jiao Tong University	China	5	0.87	136	27.20	3
13	Shenzhen University	China	5	0.87	116	23.20	5
14	Virginia Polytechnic Institute & State University	USA	5	0.87	811	162.20	4
15	Sandia National Laboratories	USA	4	0.69	263	65.75	9

**Figure 8:**
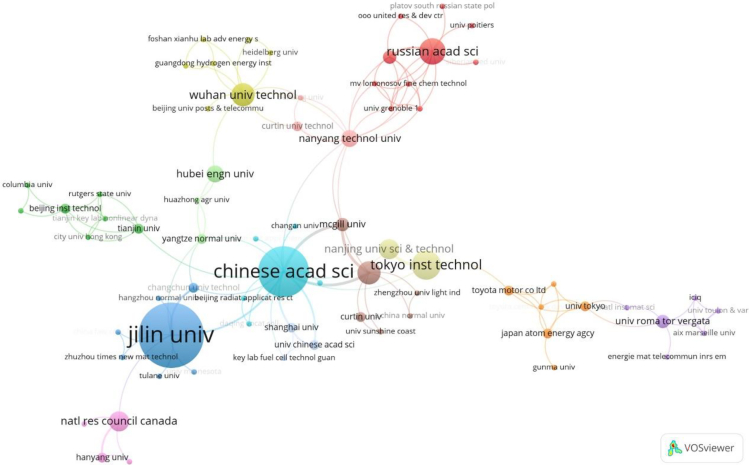
Collaboration network diagram between different institutions.

Collaboration between institutions is an effective way to promote academic communication, which can enhance academic influence, enable researchers' research results to receive more attention and recognition, and help improve academic status. By establishing a visual network of institutions, the collaborative relationship between institutions can be presented. Figure 8 shows the collaboration network map between different institutions. The nodes in the network represent different institutions, and their size depends on the number of publications of the institution. The curves represent the strength of the connection between different institutions, and the color in the visualization network represents different cluster classifications. Generally, institutions of the same color have good cooperation, while the connectivity between different colors is relatively sparse. According to the different colors, the institution’s collaborative network is divided into 13 clusters, and small clusters that are not related to each other have been removed from the network. The largest cluster (red, 10 institutions) is led by the Russian Academy of Sciences. The second cluster (blue, 7 institutions) is mainly composed of institutions in China. The second and third clusters around Jilin University and Wuhan University of Technology are mainly composed of institutions in China. It is worth noting that the Chinese Academy of Sciences plays a pivotal role in international cooperation in PEM research. In terms of connection strength, the collaboration between the Chinese Academy of Sciences and McGill University in PEM-FC research is the closest. Jilin University, the Chinese Academy of Sciences, and other institutions have jointly published many papers. In comparison, although Iran University of Science & Technology and Dalian University of Technology are relatively active institutions in PEM-FC research, their cooperation with other institutions is relatively limited. Only 70 of the 375 institutions are shown in Figure 8, indicating that collaborative research in this field is still relatively limited, and further international cooperation is needed to improve the quality of papers.

#### Author distribution and author collaboration networks

Analyzing the distribution and collaboration of paper authors can reveal the publication status of research papers and the academic influence of authors, helping researchers understand the development trends in their field and timely discover the latest research achievements. This article analyzed the authors' countries, number of published papers, paper ratios, total citations, average citations, and total links. Over the past 28 years, a total of 1,173 authors have published PEM-FC research, making them the main contributors in this field. The core authors who have published the most PEM-FC papers are shown in Table 3, and they are the top 15 most influential authors. Liu Baijun is the most prolific author, followed by Jiang Zhenhua and Ueda Mitsuru. These authors mainly come from China (43, 2.96%) and Japan (13, 0.89%), and their research in this field is significant, which is consistent with the previous research results on countries and institutions. In terms of citations, Guiver Michael D. from Canada ranks first with a total of 941 citations and an average of 188.20 citations. McGrath James E. from the United States ranks second with a total of 755 citations and an average of 188.75 citations. The paper citations of Li Xianfeng and Na Hui from China are also performing well. Liu Baijun has the highest link strength, followed by Jiang Zhenhua, indicating their strong social collaboration.

**Table 3:** Top 15 authors of published papers

Ranking	Authors	Countries	Number of documents	Proportion/%	Total citations	Citations per papers	Total link strength
1	Liu, Baijun	China	9	0.62	558	62.00	53
2	Jiang, Zhenhua	China	8	0.55	506	63.25	42
3	Ueda, Mitsuru	Japan	8	0.55	394	49.25	33
4	Li, Nanwen	China	6	0.41	166	27.67	23
5	Rowshanzamir, Soosan	Iran	6	0.41	82	13.67	13
6	Guiver, Michael D.	Canada	5	0.34	941	188.20	20
7	Higashihara, Tomoya	Japan	5	0.34	336	67.20	16
8	Abdel-hady, Esam E.	Egypt	5	0.34	33	6.60	16
9	McGrath, James E.	USA	4	0.28	755	188.75	24
10	Li, Xianfeng	China	4	0.28	652	163.00	17
11	Na, Hui	China	4	0.28	652	163.00	17
12	Jiang, San Ping	Australia	4	0.28	201	50.25	12
13	He, Gaohong	China	4	0.28	115	28.75	15
14	Liu, Hai	China	4	0.28	109	27.25	31
15	Zheng, Genwen	China	4	0.28	109	27.25	31

**Figure 9:**
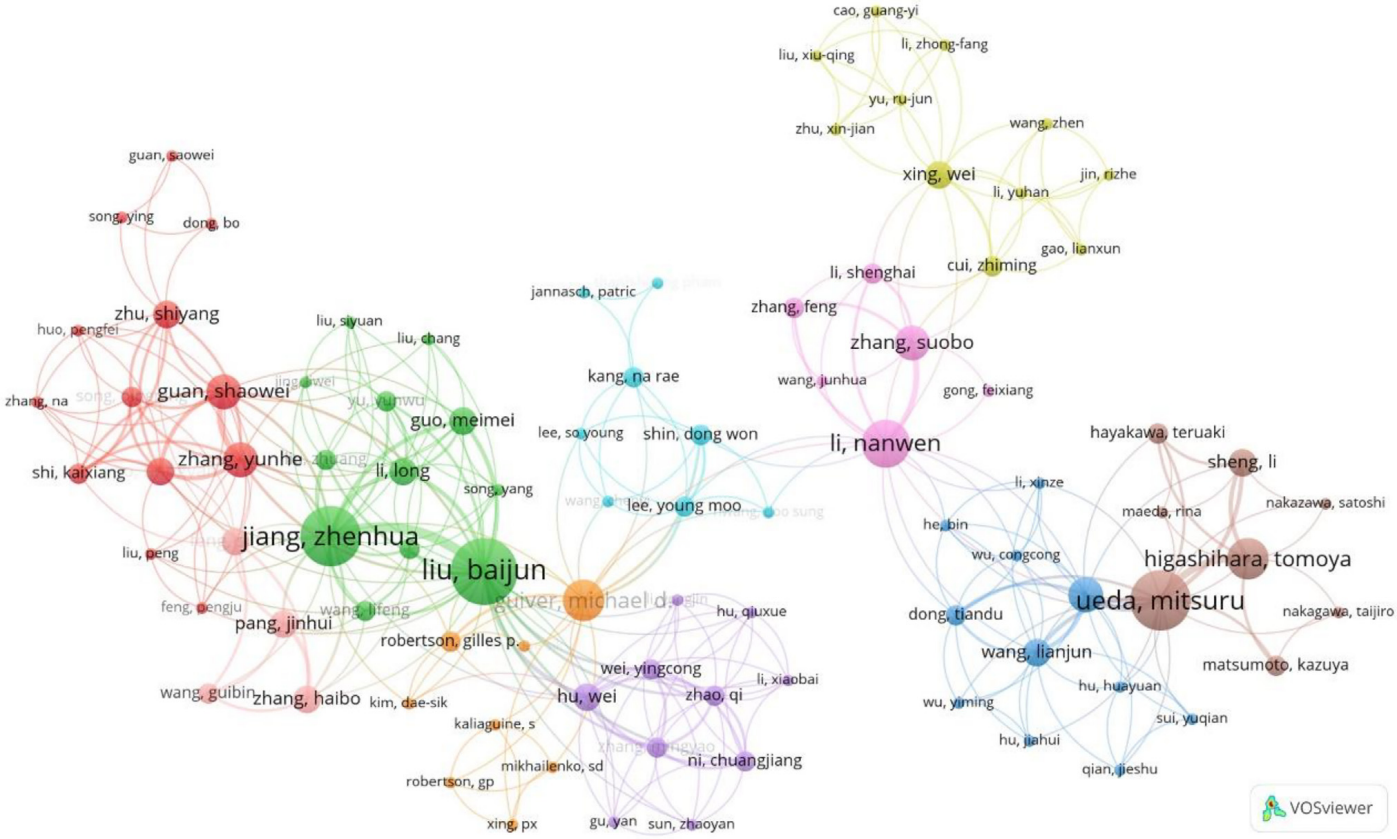
Collaborative network diagram of the authors on the PEM-FC study

To understand the different structures and scientific collaborations between institutions in this field, an interactive network graph of collaborating authors was developed, as shown in Figure 9. The circles represent authors, the colors represent clusters of authors with similar research topics, and the lines represent connections between different authors. The author collaboration network map is divided into 10 sections with different colors. The red cluster (13 authors) is mainly composed of authors from China, with Guan Shaowei and Zhang Yuehe as typical representatives. The green cluster is led by Liu Baijun and Jiang Zhenhua, with the strongest connection strength and the closest collaboration with other authors. The green cluster connects the red and purple clusters, playing a crucial bridging role in the entire PEM-FC research process. Guiver Michael D. and Li Nanwen also play important roles in this development. Zhang Xuan, Ueda Mitsuru, and Higashihara Tomoya collaborated on the research of poly(phenylene ether)s with sulfonic acid groups via long alkyl side chains as PEMs for FCs.^67^ Additionally, Liu Baijun and Guiver Michael D. conducted research on the sulfonation reaction of PEEKs with various side substituents, such as phenyl, methylphenyl, trifluoromethylphenyl, and phenoxylphenyl groups.^68^ Collaborative publication of research papers has extraordinary significance for academic exchange of ideas, enhancing academic influence, and developing research capabilities. However, the collaboration relationships in this network are relatively sparse, and there is a need to strengthen research cooperation and further densify the research network.

### Co-citation analysis

Co-citation analysis is a research methodology for assessing academic literature, which analyzes the relevance of the papers, journals, and authors of the research subject based on the co-citation relationships between them, thus helping researchers to understand the impact of academic literature as well as to identify new research directions and trends. In this section, the citation relationships of the papers, journals, and authors in the research field of PEM-FC are analyzed to facilitate a full understanding of the overall context for those interested in PEM-FC.

#### Cited references

**Figure 10:**
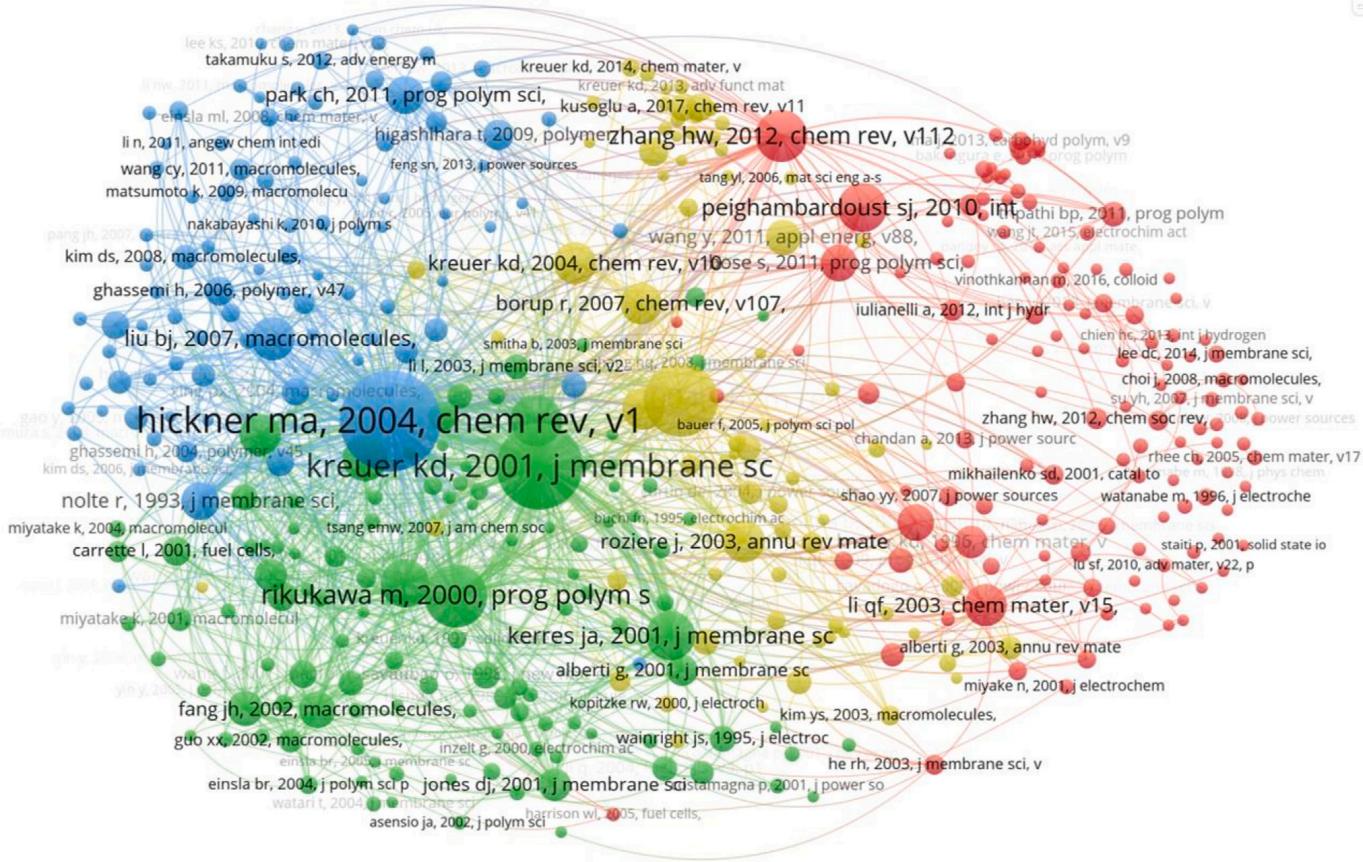
Visualization of the relationship between cited references

Citation analysis is an important means of studying knowledge in a field and can effectively track the important background of a particular study. Through literature analysis, a knowledge system can be constructed and the overall research level of the field can be evaluated. Figure 10 shows the citation relationship diagram of PEM-FC research, in which nodes represent cited literature, with their size indicating their importance, and the connecting lines representing the co-citation relationships between literatures. The circle containing Hickner, Michael A.^69^ in the blue group in Figure 10 is the largest, indicating that this study has received widespread attention from researchers and has caused a certain resonance. The connection between the literature published by Hickner, Michael A. and Kreuer Klaus-Dieter in the green group is relatively close, and the stronger the co-citation relationship between them, the closer the connection.

To specifically compare the co-citation relationships of cited literature, Table 4 lists the information of the most co-cited articles, which are the most frequently cited literature. In addition to the author, publication year, and DOI number, the table also compares the country, institution, journal, citation count, and link strength. Publications from the USA and Germany have the most citations, followed by Canada and China. These cited literatures belong to different institutions, indicating that the research topic has received wide attention from scholars around the world. Journal of Membrane Science and Chemical Reviews are the most popular journals for PEM-FC research. The literatures Hickner et al.^69^ and Kreuer^70^ have the highest citation count and link strength, and they are important literature in the knowledge system of this research field, which is consistent with the analysis results in Figure 10. They review and systematically summarize various PEMs used for FCs, analyze the transport properties and swelling behavior of different membranes, and determine the key areas for future research. Further, based on the research objectives, main findings, and limitations summarized in Table 4, we can easily understand the research background of PEM-FC, which provides significant guidance for more in-depth studies in this field.

**Table 4:** Information on the most co-cited articles

Ranking	Cited reference	First author	Country	Institution	Year	Journal	DOI	Citations	Link strength	Objectives	Key findings	Limitations
1	Hickner et al.^69^	Hickner, Michael A.	USA	Sandia National Laboratory	2004	Chemical Reviews	https://doi.org/10.1021/cr020711a	86	1594	The synthesis and proper characterization of new PEM polymer materials to meet the needs of advanced systems while maintaining a certain degree of compatibility with current hardware and system designs.	The potential of forming well-defined block copolymers can be achieved by avoiding ether-ether exchange reactions. Sulfonated high-performance polymer frameworks can be used to develop advanced PEMs. Lowering permeability seems to be a good approach to addressing the challenges of new direct methanol fuel cell (DMFC) systems.	The main obstacles are high proton conductivity under low water content conditions and long-term durability under fuel cell conditions.
2	Kreuer^70^	Kreuer Klaus-Dieter	Germany	Max-Planck-Institut für Festkörperforschung	2001	Journal of Membrane Science	https://doi.org/10.1016/s0376-7388(00)00632-3	70	1446	A comprehensive understanding of the different mechanical properties and transport characteristics of hydrated perfluorosulfonic acid polymers and low-cost sulfonated poly(arylene) under higher temperature conditions.	Based on sulfonated polyetherketone polymers, not only are they interesting low-cost alternative membrane materials for hydrogen fuel cell applications, but they also contribute to reducing issues related to high water resistance and methanol crossover in DMFCs. The conductivity is largely dependent on the migration rate of solvated protons and the structural diffusion state.	The main disadvantages faced by proton-conducting polymer membranes are high production costs, limited operating temperatures, and the crossover of water and methanol.
3	Mauritz and Moore^71^	Mauritz Kenneth A.	USA	The University of Southern Mississippi	2004	Chemical Reviews	https://doi.org/10.1021/cr0207123	57	796	The fundamental structure and properties of Nafion perfluorosulfonate materials have been analyzed to optimize their performance in applications.	The water content in Nafion has a significant impact on its performance and the hydration of cations. The static mechanical properties of Nafion can be characterized as a function of equivalent weight, counterion type, and solvent absorption. The morphology and properties of Nafion can be controlled by adjusting parameters.	The important relationship between the structure (chemical and morphological) of Nafion and its unique properties has been studied, but the understanding is not yet comprehensive. Relevant models and experimental results are limited to specific conditions of Nafion materials.
4	Rikukawa and Sanui^72^	Rikukawa Masahiro	Japan	Sophia University	2000	Progress in Polymer Science	https://doi.org/10.1016/s0079-6700(00)00032-0	46	932	This review summarizes the synthesis, chemical and electrochemical properties, as well as the application of new proton-conducting polymer electrolytes based on hydrocarbon-based polymers in fuel cells over the past decade.	Using hydrocarbon-based polymers as the polymer backbone is one of the most promising methods for preparing high-performance proton-conducting polymer electrolyte membranes.	While fluorinated polymer electrolytes exhibit satisfactory performance for successful fuel cell electrolyte membranes, the main drawbacks for large-scale commercial applications are the cost and low proton conductivity at high temperatures and low humidity.
5	Zaidi^73^	Zaidi Syed M.Javaid	Canada	Laval University	2000	Journal of Membrane Science	https://doi.org/10.1016/s0376-7388(00)00345-8	35	849	A new type of proton-conducting composite membrane has been prepared by compounding sulfonated polyether ether ketone (SPEEK) with heteropolyacids (HPA), as a potential alternative to fluorinated polymer membranes in PEMFCs.	With an increase in sulfonation degree and the introduction of fillers, the glass transition temperature and hydrophilicity of the composite membrane are enhanced, significantly improving the proton conductivity.	Further research is needed on the mechanical properties, stability, and practical application performance of the composite membrane in actual PEMFCs.
6	Zhang and Shen^74^	Zhang Hongwei	China	Sun Yat-sen University	2012	Chemical Reviews	https://doi.org/10.1021/cr200035s	35	787	The development of high-performance polymer electrolyte membranes for fuel cells has been analyzed and summarized.	The competitive cost of the nonfluorinated acidic ionomer membranes is an important development direction. Phase separation can be enhanced by appropriately increasing the length of blocks or side chains. Cross-linkable groups can be attached to the membrane to improve the expansibility and durability of PEM.	Developing cheaper and more efficient PEMs still presents certain challenges.
7	Kerres^75^	Kerres Jochen A.	Germany	University of Stuttgart	2001	Journal of Membrane Science	https://doi.org/10.1016/s0376-7388(00)00631-1	33	743	The latest developments in proton-conducting polymer (composite material) membranes for fuel cells are outlined, particularly in the field of chemical engineering.	Covalently crosslinked ionomer membranes exhibit high-temperature dimensional stability. In particular, type I covalently crosslinked membranes, in which all macromolecules participate in a covalent network, demonstrate high-dimensional stability. The main advantages of ionically crosslinked ionomer membranes are high mechanical flexibility and good mechanical stability.	Due to the rigid covalent network, covalently crosslinked ionomer membranes exhibit brittleness in a dry state. In ionically crosslinked acid-base blends, the breaking of ionic crosslinks may lead to insufficient membrane size stability.
8	Peighambardoust et al.^76^	Peighambardoust Jamaleddin Seyed	Iran	Iran University of Science and Technology	2010	International Journal of Hydrogen Energy	https://doi.org/10.1016/j.ijhydene.2010.05.017	33	558	A proton exchange membrane with high proton conductivity, low electron conductivity, low fuel permeability, low electroosmotic drag coefficient, good chemical/thermal stability, good mechanical properties, and low cost.	The complexity of PEMFC systems can be reduced by developing "water-free" electrolytes that do not require hydration. Strategies to replace PEM include modified Nafion composite membranes, functionalized nonfluorinated membranes and their composites, acid-base composite membranes.	Current PEMFC technology is based on expensive perfluorinated PEMs that only work effectively under fully hydrated conditions. The development of various membranes has made it difficult to simultaneously meet the performance requirements of fuel cells.
9	Wang^77^	Wang Feng	USA	Virginia Polytechnic Institute and State University	2002	Journal of Membrane Science	https://doi.org/10.1016/s0376-7388(01)00620-2	32	728	Developing poly(arylene ether sulfone)s with high water uptake and good stability may lead to new polymer electrolyte materials for PEMFC.	The electrical conductivity is greatly influenced by ion exchange capacity, temperature, and water activity. The molar ratio of sulfonation reaction significantly impacts the membrane’s electrical conductivity and water swelling ratio.	The performance of highly sulfonated copolymers in fuel cells requires further testing. Additionally, comprehensive systematic studies, such as high-temperature conductivity, are also needed for further research.
10	Xing^78^	Xing Peixiang	Canada	National Research Council	2004	Journal of Membrane Science	https://doi.org/10.1016/j.memsci.2003.09.019	29	562	The sulfonated poly(ether ether ketone)s (SPEEKs) were prepared by sulfonating Victrex® and Gatone® PEEK, and their performance was compared with that of PEM.	Elevated temperatures did not cause any significant chain degradation in Victrex® and Gatone® PEEK. The membrane conductivity increased with the degree of sulfonation and temperature.	The relevant experimental results are greatly influenced by specific external environmental factors.
11	Liu et al.^68^	Liu Baijun	China	Jilin University	2007	Macromolecules	https://doi.org/10.1021/ma061705	28	646	Developing aromatic poly(ether ketone)s (PEKs) with pendant sulfonic acid phenyl groups for PEMs.	The presence of different pendant groups can facilitate rapid postsulfonation under mild reaction conditions. A range of PEKs can be obtained by controlling the length of the unsulfonated segments in homopolymers and copolymers.	It is challenging to precisely control experimental conditions to obtain high-performance PEM materials.
12	Kreuer et al.^79^	Kreuer Klaus-Dieter	Germany	University of Alabama in Huntsville	2004	Chemical Reviews	https://doi.org/10.1021/cr020715f	27	502	The best current understanding of mass and charge transport in proton conductors related to fuel cells is provided, including a description of the fundamental reactions involved in these transport processes and a quantitative phenomenological description of transport in these materials.	Understanding transport properties as a function of molecular structure and morphology and the development of new separation materials are the current focus of fuel cell research. Simulation techniques should be appropriately combined with experimental results to establish improved and new pathways for proton conducting materials.	The treatment of transport properties of proton conducting materials often relies on macroscopic approaches and controversial transport mechanisms.
13	Li et al.^80^	Li Qingfeng	Denmark	Technical University of Denmark	2003	Chemistry of Materials	https://doi.org/10.1021/cm0310519	27	616	The research methods, potential systems, and current applications of polymer electrolyte membranes for fuel cells operating at temperatures above 100°C were analyzed.	Improved PFSA membranes have been successfully used in DMFC and H_2_/O_2_(air) cells operating at high temperatures and pressures. Sulfonated hydrocarbons and their inorganic composite materials have shown good conductivity and thermal stability at temperatures above 100°C. Acid-base polymer membranes have been proven to operate at temperatures as high as 200°C in DMFC and H_2_/O_2_ (air) PEMFC.	Some key issues and drawbacks of PEMFC technology based on perfluorosulfonic acid (PFSA) include water management, carbon monoxide poisoning, hydrogen, reformate salts, and methanol as fuel, as well as cooling and heat recovery.
14	Rozière^81^	Rozière Jacques	France	Université Montpellier II	2003	Annual Review of Materials Research	https://doi.org/10.1146/annurev.matsci.33.022702.154657	27	683	The developed fluorine-free polymer membranes exhibit sufficient mechanical strength at functionalization and hydration levels, high proton conductivity, and stability in harsh environments, particularly in terms of thermal and chemical stability.	The efficiency of fuel cells is a function of their power density during operation, and the optimum nominal efficiency depends on the performance and capital cost of the fuel cell. The role of polymer membranes in fuel cells is proton conduction and gas separation. Fluorine-free polymer membranes can serve as potential candidate materials for PEM and DMFC.	Although some fluorine-free polymer membranes have been proven to have a lifespan of 500–4000 h, there are few systems that can operate long-term above 100°C.
15	Steele and Heinzel^82^	Steele Brian C. H.	UK	Imperial College	2001	Nature	https://doi.org/10.1038/35104620	27	566	The research progress of innovative alternative materials for fuel cell technology was summarized.	The comprehensive area-specific resistance of battery components should be less than 0.5 Vcm^2^ to ensure high power density. The main materials used for the development of PEMFC stacks include ion conducting membranes, electrocatalysts, cell frames, and bipolar plates.	The main issues that need to be addressed for the market application of fuel cell technology include the optimal selection of fuel and the development of alternative materials for fuel cell stacks.

#### Cited sources

**Figure 11:**
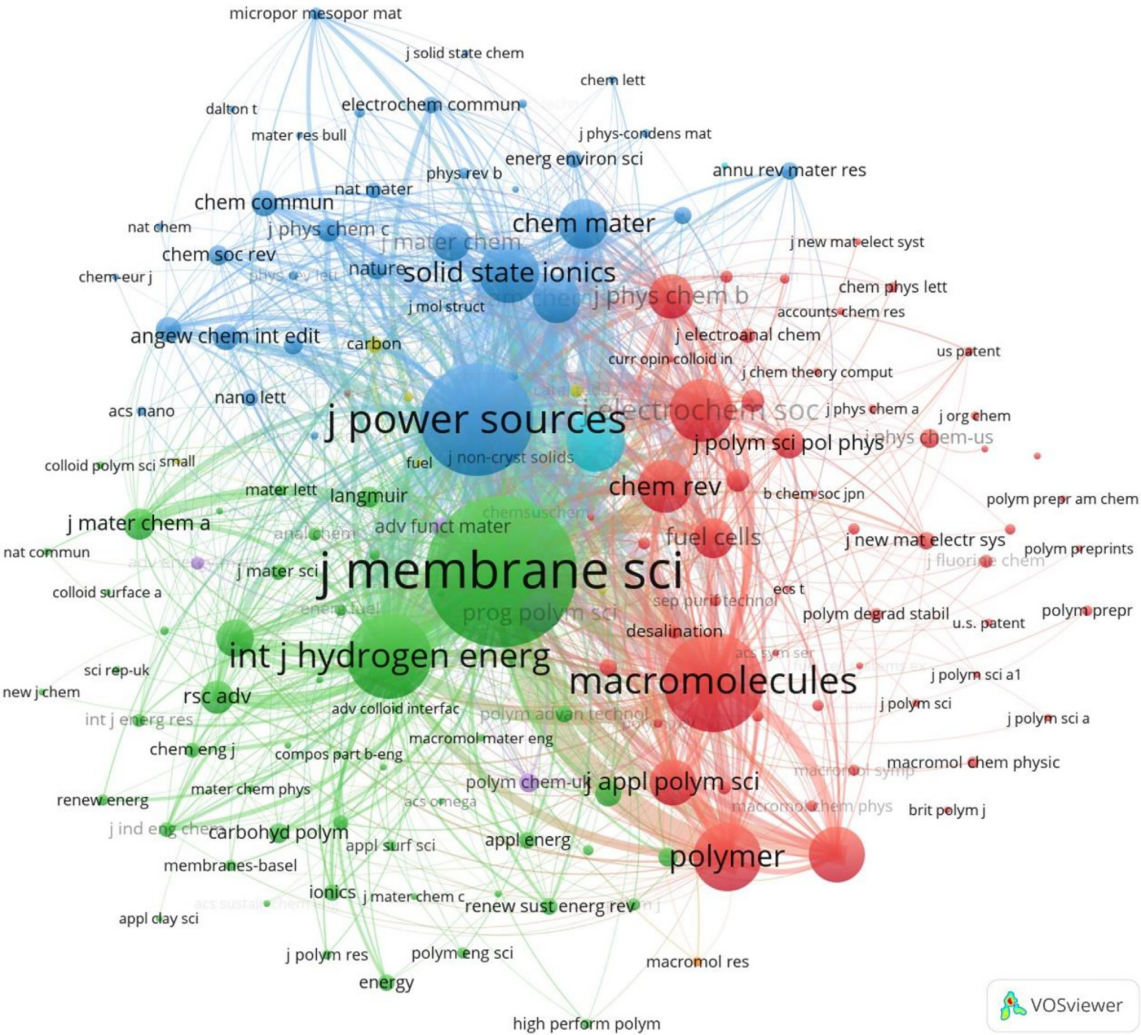
Visualization of relationships for journal co-citation analysis

Journal co-citation analysis is a social network analysis method for studying academic literature, which can establish connections between various journals and explore the influence and contribution of academic literature. By analyzing the citation relationships of journals, important journals in the research field can be identified, hot issues and research directions in the academic community can be traced, existing knowledge networks can be explored, and the most influential journals can be identified. The cited sources of 301 research papers come from 1,580 journals. Setting the minimum citation frequency of a journal to 10, Figure 11 shows the visualized co-citation relationship of 157 journals. The connections between nodes indicate the citation links between journals. Journal of Membrane Science leads the entire green cluster, and its node is the largest; its co-citation relationship with other journals is also the closest. The most active journals shown in Table 5, Journal of Membrane Science, has a co-citation frequency and link strength of 1,873 and 147,153, respectively, indicating that it is the core journal in the PEM-FC field. Journal of Power Sources and Macromolecules rank second and third, respectively, in terms of citation volume. The organic combination relationship between these journals reveals the interdependence and cross-knowledge system in the PEM field. According to the classification method of ESI on subject categories, there are 11 chemistry, 2 materials science, 1 engineering, and 1 physics. It can be seen that the co-citation analysis of journals can determine the disciplinary properties and scientific structure of journals, identify the core areas of disciplines, and effectively evaluate academic journals.

**Table 5:** Information on the most co-cited journals

Ranking	Cited sources	Subject category	Citations	Link strength
1	Journal of Membrane Science	Chemistry	1873	147153
2	Journal of Power Sources	Materials science	1143	107320
3	Macromolecules	Chemistry	932	54460
4	International Journal of Hydrogen Energy	Engineering	720	70697
5	Polymer	Chemistry	497	32173
6	Journal of the Electrochemical Society	Chemistry	439	34998
7	Solid State Ionics	Physics	407	47547
8	Electrochimica Acta	Chemistry	391	39194
9	Journal of Polymer Science Part A Polymer Chemistry	Chemistry	355	20856
10	Chemical Reviews	Chemistry	329	19882
11	Chemistry of Materials	Materials science	298	32411
12	Journal of the American Chemical Society	Chemistry	270	26593
13	Journal of Applied Polymer Science	Chemistry	267	15978
14	Journal of Physical Chemistry B	Chemistry	246	21067
15	Fuel Cells	Chemistry	211	16820

#### Cited authors

**Figure 12:**
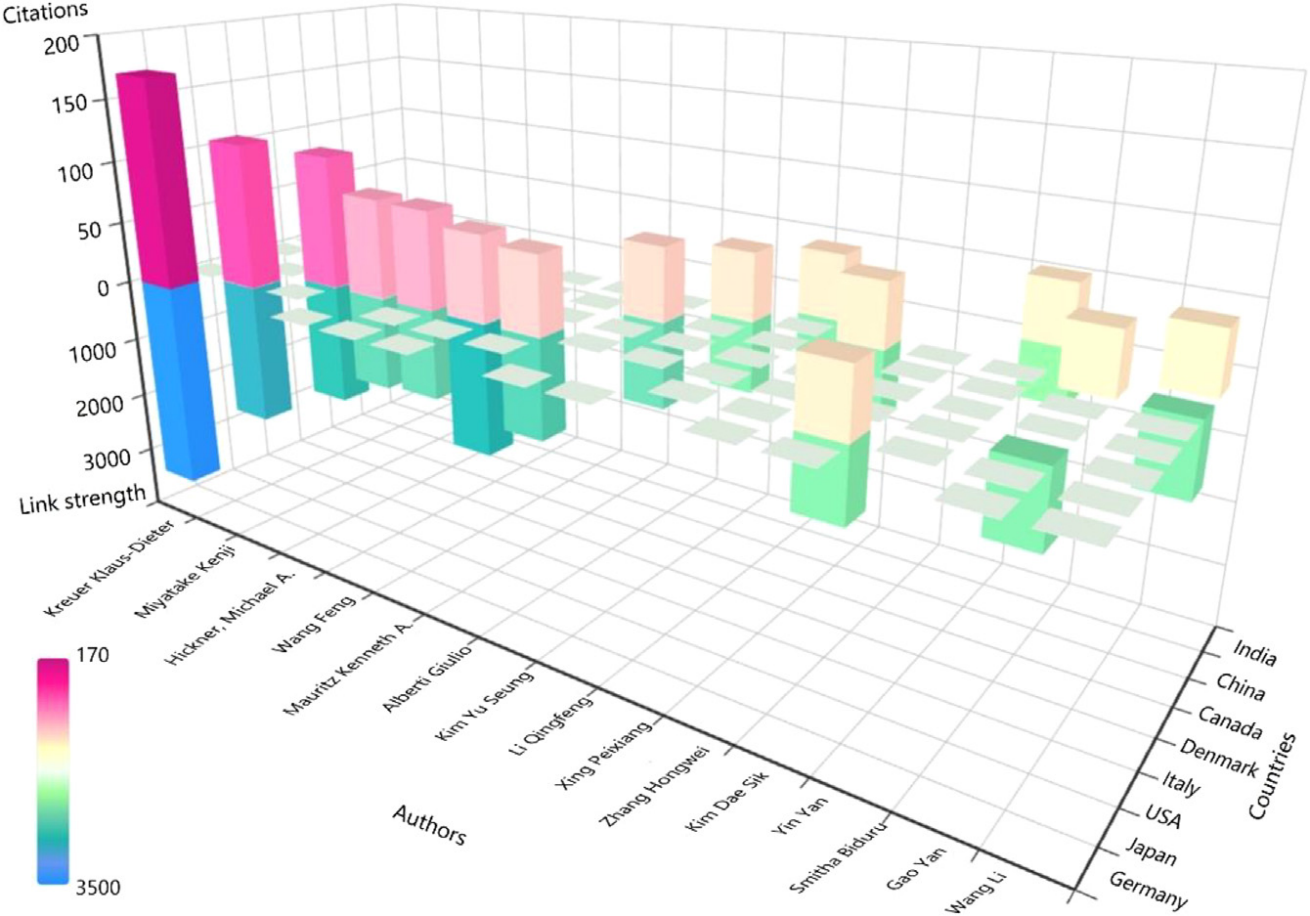
Distribution of authors with the highest number of citations in the co-citation analysis

**Figure 13:**
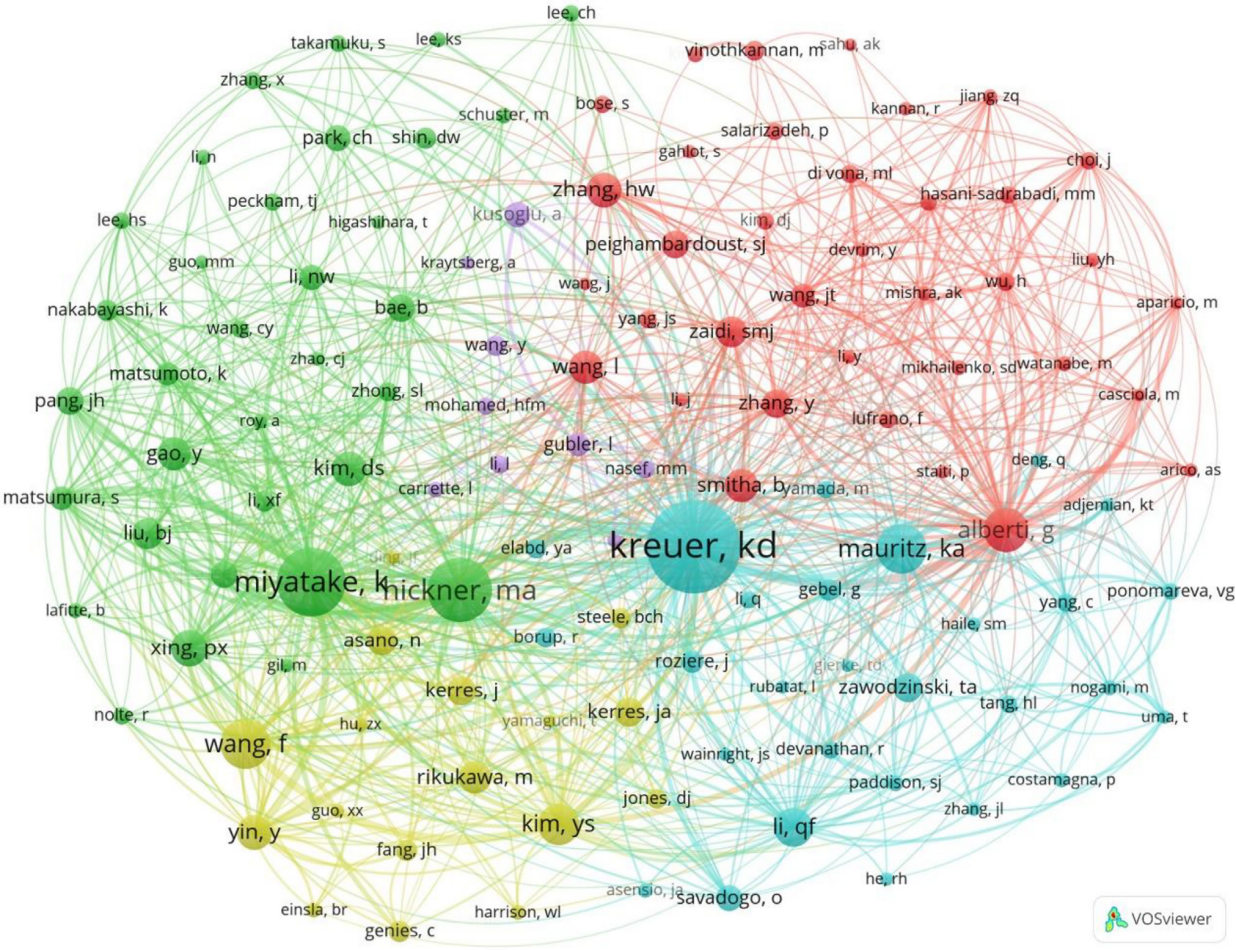
Visualization of the relationship between authors in the co-citation analysis

Author co-citation analysis can reflect the academic influence of an author and help us understand the academic level and development trends of their research, providing accurate reference for other researchers. The development of a discipline relies heavily on the support of outstanding scholars. Figure 12 shows the top 15 authors with the highest co-citation counts. Many authors are from the United States, followed by Canada and China. Klaus-Dieter Kreuer ranks first with 170 citations and a link strength of 3,412, followed by Kenji Miyatake and Michael A. Hickner. These authors have made significant contributions to FCs, driving scientific and technological advancements, and having a profound impact on future research. A total of 6,063 authors participated in research in this field, with a minimum citation count of 15. Visual analysis was conducted on 123 cited authors, as shown in Figure 13. Nodes represent authors, and, the larger the node, the more citations the author has in the network, indicating greater influence, as seen in the cases of Klaus-Dieter Kreuer and Michael A. Hickner. The red cluster is led by Giulio Alberti from Italy, with 36 co-citation connections among them. The well-distributed nodes promote positive development in the scientific field and reflect the interdisciplinary nature of the research.

### Co-occurrence and clustering analysis

Co-occurrence analysis is a text analysis method that identifies and calculates a group of keywords that appear together in a set of texts. It aims to discover the relationships and mutual influences between these keywords, revealing hidden information and underlying patterns in the text. Co-occurrence analysis is of great significance as it not only identifies the themes of the text, helping people better understand the content and key information, but also reveals the potential relationships between keywords, including both related and relatively independent relationships, providing insightful information such as research topics and hotspots. And cluster analysis is a multivariate data analysis method that aims to group similar observational units together, forming different clusters. It helps people better understand the patterns and features in a dataset, transforming messy data structures into useful information and knowledge. By employing cluster analysis, people can quickly identify patterns and trends in large amounts of data, uncovering the underlying patterns behind the data, and providing important evidence for decision-making. Additionally, the differences and similarities between different clusters can be clearly expressed, providing new entry points and ideas for further exploration.

**Figure 14:**
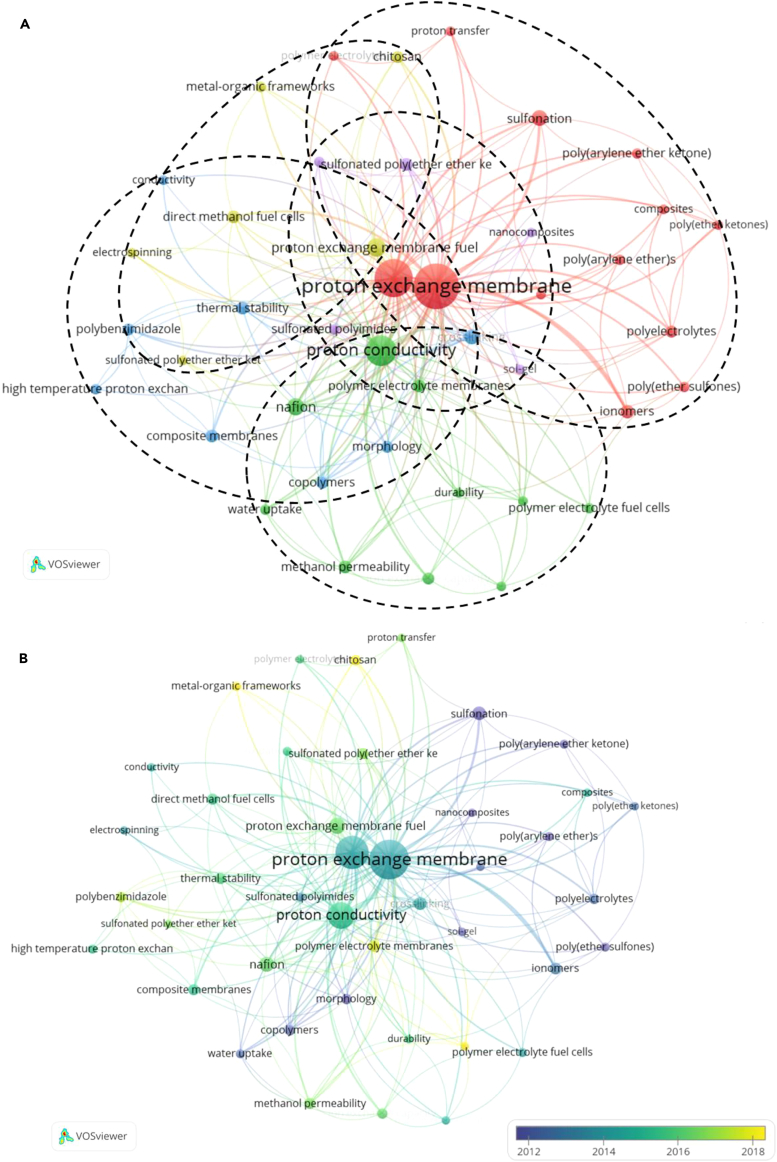
Co-occurrence and clustering analysis of PEMs studies (A) Co-occurrence network map. (B) Time evolution map.

After merging and eliminating 631 keywords used in the research of PEM-FCs, 558 keywords remained. By selecting keywords with a co-occurrence frequency greater than 4, it was found that a total of 42 keywords reached the threshold. A correlation score was calculated for these keywords, and the most relevant keywords were selected to create a map, as shown in Figure 14A. Each circle represents a keyword, and the size of the circle represents the frequency of occurrence of that keyword. The strength of the connections between these keywords is represented by the width of the lines connecting the circles. Based on the co-occurrence relationships between keywords, it is evident that Figure 14A is divided into five clusters, represented by red, green, blue, yellow, and purple.

In the red cluster, there are 13 keywords, with "proton exchange membrane" appearing most frequently, with a total link strength of 222. The main keywords also include fuel cells, ionomers, phase separation, poly(arylene ether ketone), poly(arylene ether), poly(ether ketones), poly(ether sulfones), polyelectrolytes, polymer electrolytes, proton transfer, sulfonation, and composites. These clustered keywords mainly relate to research on PEMs and FCs, emphasizing the importance of PEMs in FC applications. Ref.^83^^,^^84^^,^^85^^,^^86^ provide comprehensive explanations on this point. The green cluster has the second highest number of keywords, primarily focusing on proton conductivity. This cluster consists of keywords such as durability, ion exchange capacity, mechanical properties, methanol permeability, microphase separation, Nafion, polymer electrolyte fuel cells, polymer electrolyte membranes, and water uptake. These keywords are related to PEMs and their relevant properties, highlighting the necessity of understanding PEM performance. This can be seen in ref. ^87^^,^^88^^,^^89^^,^^90^^,^^91^. The blue cluster mainly includes 8 keywords: composite membrane, conductivity, copolymers, crosslinking, high temperature PEM, morphology, polybenzimidazole, and thermal stability. Some of these technical terms indicate that the blue cluster is related to material modification and enhancement mechanisms,^92^ focusing on improving the performance of PEMs. Relevant research can be found in ref. ^93^^,^^94^^,^^95^^,^^96^^,^^97^^,^^98^. The yellow and purple clusters consist of 6 and 5 keywords, respectively, focusing on the preparation processes and methods of PEMs, as well as the development of emerging materials such as polyoxadiazoles,^99^ sulfonated PI,^100^^,^^101^ MoS_2_-NiO-Co_3_O_4_ filled chitosan,^102^ and branched polymer materials.^103^ In summary, the different clusters represent research on different aspects of PEMs, including materials, properties, mechanisms, and optimization. This lays the foundation for their application in FCs.

Figure 14B is a time-varying deduction of the mapping network of PEMs. The color of the keywords in the figure represents the average year of publication. It can be observed that earlier research by most scientists mainly focused on the unique properties of the exchange membrane itself, such as sulfonation, poly(arylene ether ketone), polyelectrolytes, copolymers, morphology, and water uptake. Subsequently, there was a widespread study of PEMs and PEMFCs, such as PEM, ionomers, FCs, conductivity, electrospinning, and high-temperature PEM. These studies illustrate the important role and related mechanisms of PEMs in FCs. In recent years, based on the research foundation of PEMs, people have begun to focus on the development of more efficient and stable new PEMs, further emphasizing their practical significance in sustainable energy applications and environmental protection. Relevant terms include metal-organic frameworks, polybenzimidazole, polymer electrolyte membranes, chitosan, and mechanical properties. The research hotspots in different periods also reflect the process of people’s understanding of PEMs changing. By analyzing the inherent relationships of keywords, the study reveals their role in enhancing FC performance and enhancing strategies.

In summary, the performance of PEMs has a significant impact on FC performance and application. With the development of science and technology, some requirements have been proposed for PEMs used in FCs, which are as follows. (1) High proton conductivity: the proton conductivity of the PEM directly affects the efficiency of the FC. High proton conductivity can ensure that protons can be quickly transported within the membrane,^104^ thereby improving the output power and efficiency of the cell. (2) Low electronic conductivity: lower electronic conductivity can prevent electron leakage and reduce the loss of electrochemical reactions. (3) Good chemical stability: PEMs should have good chemical stability to avoid corrosion or degradation in FCs.^105^^,^^106^ Poorly chemically stable PEMs may be affected by fuel and oxygen, leading to membrane degradation, affecting the life and stability of FCs. (4) Appropriate thermal stability: FCs generate high temperatures during operation, so PEMs need to have good thermal stability to avoid failure at high temperatures.^107^ Poorly thermally stable PEMs may deform, rupture, or lose proton conductivity, affecting the performance and life of FCs. (5) Appropriate water management capabilities: PEMs should have appropriate water management capabilities,^108^^,^^109^^,^^110^ able to absorb and release appropriate amounts of water during FC operation to maintain the moist state of the membrane and ensure smooth proton conduction.^111^^,^^112^^,^^113^ (6) Good mechanical strength and flexibility: PEMs need to withstand the vibration and deformation of the FC system while maintaining the continuity of proton conduction channels.^114^^,^^115^ (7) Good durability: poorly durable PEMs may be affected by factors such as oxidation, degradation, and corrosion,^116^^,^^117^ leading to membrane failure, affecting the life and performance stability of FCs. Therefore, PEMs with good performance can ensure the high efficiency, long life, and stable operation of FC systems. Future development will inevitably require the intelligent and sustainable development of materials.

## Challenges and potential developments in PEMs

**Figure 15:**
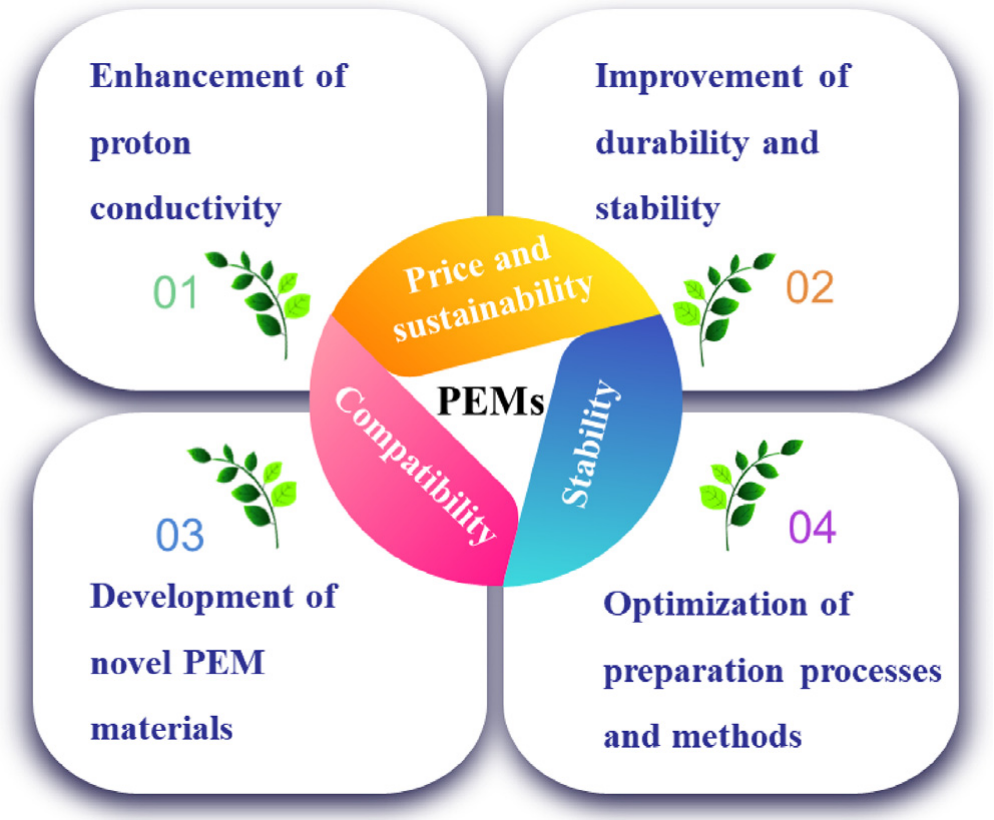
Challenges and development directions for PEMs used in FCs

The PEM is a core component of FCs and directly affects their performance and stability. Therefore, research on PEM has significant theoretical and practical significance. Figure 15 presents the main challenges and development directions in the research of PEM-FCs.

Currently, the main challenges faced by PEMs are listed as follows:

(1) Price and sustainability of PEMs

The price of PEMs is an important challenge that hinders the commercial application of FC technology. Traditional PEM materials, such as polytetrafluoroethylene (PTFE), are expensive and have high manufacturing costs. One solution to this problem is to develop low-cost alternative materials, such as polystyrene and PEEK. These materials have lower prices and can be obtained through simple manufacturing processes. Additionally, improving the lifespan and stability of PEMs can reduce the maintenance and replacement costs of FC systems, thereby lowering overall costs.

(2) Stability of PEMs in high-temperature and high-humidity environments

The PEMs are prone to degradation and failure in high-temperature and high-humidity environments, which limits the operating temperature and humidity range of FCs. Developing PEM materials with better thermal stability and moisture resistance is key to addressing this issue. For example, organic-inorganic hybrid materials exhibit good thermal and humidity stability and can be used to prepare PEMs with higher operating temperature and humidity ranges. Furthermore, optimizing the structure and morphology of PEMs, such as increasing crosslinking and adjusting porosity, can enhance their stability.

(3) Compatibility of PEMs with other components

The PEMs need to have good contact and compatibility with other components, such as electrodes, to ensure the normal operation of FC systems. However, compatibility issues between different materials can lead to poor contact and hindered electron conduction. Developing PEM materials with good compatibility is crucial. For example, adjusting the surface properties of PEMs, such as introducing specific functional groups and altering the material’s hydrophilicity, can improve their compatibility with other components.

The performance and stability of PEMs directly impact the efficiency and lifespan of FCs. Therefore, improving the proton conductivity, durability, and stability of PEMs, as well as developing new PEM materials, is an important research direction in the field of PEMs. Specific research directions for the future can be explored in the following aspects:

(1) Enhancement of proton conductivity of PEMs

Proton conductivity is one of the key indicators of PEMs, directly determining the power output and efficiency of FCs. Currently, some commonly used PEMs already have high proton conductivity, but there are still limitations. Therefore, researchers are actively searching for new proton exchange groups and improving the conduction mechanisms to enhance the proton conductivity of PEMs. For example, introducing new proton exchange groups such as phosphoric acid groups and boronic acid groups can enhance the proton conductivity of PEMs. Additionally, optimizing the structure and morphology of PEMs, such as adjusting the porosity of the membrane and increasing the concentration of proton exchange groups, can also improve proton conductivity.

(2) Improvement of durability and stability of PEMs

During long-term use, PEMs can be affected by factors such as oxidation, hydrolysis, and dehydration, leading to performance degradation or even failure. Therefore, improving the durability and stability of PEMs is a key focus of research. One approach is to improve the material and structure of PEMs to enhance their resistance to oxidation, hydrolysis, and dehydration. Another approach is to develop new PEM materials with better stability, such as organic-inorganic hybrid materials and polymer composites. These materials exhibit improved thermal stability and durability, which can enhance the lifespan and stability of PEMs.

(3) Development of novel PEM materials

Currently, commonly used PEM materials include PTFE, polystyrene, and PEEK. However, these materials still have limitations in terms of proton conductivity, durability, and stability. Therefore, researchers are actively developing novel PEM materials. For example, organic-inorganic hybrid materials combine the advantages of organic polymers and inorganic materials, exhibiting high proton conductivity and stability. Polymer composites, by combining the PEM with other functional materials such as nanoparticles and carbon nanotubes, can further enhance the performance and functionality of PEMs.

(4) Optimization of preparation processes and methods for PEMs

The preparation processes and methods of PEMs have a significant impact on their performance and stability. Therefore, optimizing the preparation processes and methods for PEMs is a key aspect of research. For example, using different polymerization methods, adjusting reaction conditions, and controlling the degree of crosslinking can influence the structure, morphology, and distribution of proton exchange groups in PEMs. Additionally, employing new preparation methods such as solution impregnation and membrane casting can improve the efficiency and consistency of PEM preparation.

## Conclusion

The research progress and application prospect of PEM in FCs are exciting. As a key component of FCs, PEM plays a crucial role in the performance and stability of FCs. This article systematically analyzes and summarizes the research progress of PEM-FCs and gives an outlook on the current challenges and future development directions. There are some challenges to overcome for PEMs, such as price and sustainability issues, stability issues in high-temperature and high-humidity environments, and compatibility issues with other components. Improving the proton conductivity, durability, and stability of PEMs, as well as developing new PEM materials and optimizing the preparation processes and methods of PEMs, is an important direction for PEM research. Further improving the performance of PEMs through continuous innovation and research will promote the development of FC technology and contribute to the application of sustainable energy and environmental protection.

## Data and code availability

No data were used for the research described in the article.
